# Change in the coding of interaural time difference along the tonotopic axis of the chicken nucleus laminaris

**DOI:** 10.3389/fncir.2015.00043

**Published:** 2015-08-20

**Authors:** Nicolas Palanca-Castan, Christine Köppl

**Affiliations:** Cluster of Excellence “Hearing4all” and Research Center Neurosensory Science and Department of Neuroscience, School of Medicine and Health Sciences, Carl von Ossietzky University of OldenburgOldenburg, Germany

**Keywords:** interaural time differences, chickens, auditory brainstem, nucleus laminaris, *in vivo* electrophysiology

## Abstract

Interaural time differences (ITDs) are an important cue for the localization of sounds in azimuthal space. Both birds and mammals have specialized, tonotopically organized nuclei in the brain stem for the processing of ITD: medial superior olive in mammals and nucleus laminaris (NL) in birds. The specific way in which ITDs are derived was long assumed to conform to a delay-line model in which arrays of systematically arranged cells create a representation of auditory space with different cells responding maximally to specific ITDs. This model was supported by data from barn owl NL taken from regions above 3 kHz and from chicken above 1 kHz. However, data from mammals often do not show defining features of the Jeffress model such as a systematic topographic representation of best ITDs or the presence of axonal delay lines, and an alternative has been proposed in which neurons are not topographically arranged with respect to ITD and coding occurs through the assessment of the overall response of two large neuron populations, one in each hemisphere. Modeling studies have suggested that the presence of different coding systems could be related to the animal’s head size and frequency range rather than their phylogenetic group. Testing this hypothesis requires data from across the tonotopic range of both birds and mammals. The aim of this study was to obtain *in vivo* recordings from neurons in the low-frequency range (<1000 Hz) of chicken NL. Our data argues for the presence of a modified Jeffress system that uses the slopes of ITD-selective response functions instead of their peaks to topographically represent ITD at mid- to high frequencies. At low frequencies, below several 100 Hz, the data did not support any current model of ITD coding. This is different to what was previously shown in the barn owl and suggests that constraints in optimal ITD processing may be associated with the particular demands on sound localization determined by the animal’s ecological niche in the same way as other perceptual systems such as field of best vision.

## Introduction

Interaural time differences (ITDs) are the small differences in the arrival of a sound at the two ears of an animal. These ITDs are used by the brain to determine the origin of a sound in the horizontal plane. The presence of temporally very precise processing mechanisms enables animals to detect very small ITDs and to discriminate between ITDs separated by only a few microseconds, using them to encode sound source location in the azimuthal plane (Joris and Yin, 2007). ITD computing is first carried out in specific, tonotopically organized areas of the brainstem nucleus laminaris (NL) in birds and the medial superior olive (MSO) in mammals. Neurons in these nuclei act as coincidence detectors, firing maximally when the phase of the inputs from both ears is the same (Goldberg and Brown, 1969; Carr and Konishi, 1990; Yin and Chan, 1990).

Coincidence detection is combined with mechanisms that delay inputs from one side, resulting in the “tuning” of these neurons to the specific acoustic ITD that compensates for the neural input delay. This value, termed “best ITD,” will elicit a maximal response from the neuron. Several possible mechanisms for creating delays have been proposed (reviewed in Vonderschen and Wagner, 2014). These mechanisms include differences in length and/or myelination of input axons (Jeffress, 1948; Cheng and Carr, 2007; Seidl et al., 2010, 2014), precisely timed inhibition (Brand et al., 2002; Grothe et al., 2010), cochlear delays (Shamma et al., 1989; Day and Semple, 2011), asymmetric synaptic rise times (Jercog et al., 2010), asymmetric spectrotemporal tuning of left and right inputs (Fischer et al., 2011) and dynamic changes at the coincidence detection stage itself (Franken et al., 2015). Data from birds and crocodilians (archosaurs) support a system of axonal time delay lines, as first suggested by Jeffress (1948) that creates a topographic array of NL neurons, each responding maximally to sounds from a specific ITD. Together, these form a map or place code of azimuthal space (reviewed in Ashida and Carr, 2011).

Data from mammals support an alternative model, in which neurons from a given frequency band in MSO respond maximally to a contralaterally leading ITD that lies outside the naturally heard range (defined by the animal’s head size). This places the slope, rather than the peak, of the response curve into the natural ITD range and the derivation of a specific azimuthal location then requires the comparison of activity levels between the two brain hemispheres. This “two-channel model” represents a population code and has been suggested to rely on phase delays created through precisely timed inhibition (reviewed in Grothe et al., 2010). However, the existence of sufficiently precise, phase-locked inhibition is controversial (Zhou et al., 2005; Roberts et al., 2013) and recent evidence suggests that removal of glycinergic inhibition in gerbil MSO brain slices has no systematic effect on the ITD tuning of neurons (Franken et al., 2015).

The intuitive conclusion from these findings is that archosaurs and mammals have evolved different ITD-processing mechanisms. However, work by Harper and McAlpine (2004) and Harper et al. (2014) on optimal ITD-coding strategies opened up a different interpretation, suggesting that animal head size and the frequency range of coding may be the primary factors that determine which neural code is used. Although more robust coding models dealing with more natural stimuli have also been put forward, models do consistently differ in performance with frequency and head size (Goodman et al., 2013).

Previous studies of chicken NL (Köppl and Carr, 2008) suggested that the distribution of best ITDs at low characteristic frequencies (below 1000 Hz) is different than at higher frequencies. Higher frequencies had a contralaterally biased best ITD distribution consistent with observations in mammals, alligators and the barn owl (Sullivan and Konishi, 1986; Carr and Konishi, 1990; McAlpine et al., 2001; Peña et al., 2001; Brand et al., 2002; Carr et al., 2009; Palanca-Castan and Köppl, 2015). In contrast, lower frequencies contained a similar number of neurons coding ipsi- and contralaterally leading ITDs, indicating a possible change in the ITD-coding strategy with frequency, as in principle suggested before (Harper and McAlpine, 2004; Harper et al., 2014). More recently, Fischer and Seidl (2014) put forward the specific prediction that the peaks of ITD-response functions cease to be informative below 500 Hz, at which point they become broader than the physiological range of the chicken; a given neuron would then fire maximally in response to any sound regardless of the position of its source in azimuthal space. The authors argue that under these conditions a Jeffress-like place code would not allow to localize sounds sources effectively and suggest that if chickens are able to localize frequencies below 500 Hz, the coding system at those frequencies should be based on the slope of the response curve. Note that such a slope code does not necessarily equate with a change to a two channel coding model. Instead, a variation of Jeffress’ place code could be implemented, based on the slopes instead of the peaks of response functions, as suggested by Hyson (2005). Unfortunately, there are currently no behavioral data regarding the chicken’s sound localization ability at any frequency.

The aim of the present study was to collect a comprehensive dataset of physiological responses of chicken NL neurons tuned to frequencies of 1500 Hz and below that could then be compared with mammalian data, which usually lie within that same frequency range. In addition, we were especially interested to confirm or refute the presence of a symmetrical best ITD distribution at lower frequencies. Lastly, we were interested in testing the prediction of a change to a slope-code system at frequencies below 500 Hz.

Our results differed considerably from a companion study in the barn owl (Palanca-Castan and Köppl, 2015) and point to a change in the ITD coding system of the chicken at the lower frequencies.

## Materials and Methods

### Experimental Animals and Preparation

We report data from 25 chickens (*Gallus gallus*) of both sexes and aged between 3 and 7 weeks. Peripheral responses at the level of the auditory nerve are largely mature at hatching and fully mature at 3 weeks of age (Manley et al., 1991). Similarly, the synaptic connections and adult cell morphology of neurons in nucleus magnocellularis and NL are essentially present at hatching and fully mature around 3–4 weeks of age (reviewed in Kubke and Carr, 2005). Physiologically, maturation is also likely to extend beyond hatching (Kuba et al., 2002) but the endpoint has not been explored in any detail.

Seven chickens were of the wild-type *Gallus gallus bankiva* subspecies, the remaining ones of various commercial *Gallus gallus domesticus* breeds: three White Leghorn from pathogen-free eggs, and 15 Lohmann Braun. Bankiva chickens originated from a breeding colony at the animal facility of the University of Oldenburg; White Leghorns were hatched and raised at the same facility; Lohmann Braun were acquired from a commercial breeder at the day of hatching and also raised at the same facility. Therefore, although different breeds were sourced from different providers, all chickens were exposed to the same environment after hatching. We tested each of the examined parameters for differences between data taken for different breeds; Kruskal–Wallis test, *p* = 0.616 for best ITD, *p* = 0.405 for best interaural phase difference (IPD), *p* = 0.068 for characteristic delay (CD), and *p* = 0.303 for characteristic phase (CP). There were no significant differences in the data from Bankiva (wild type) chickens and breeds originating from either labs or commercial breeders (White leghorn and Lohmann Braun, respectively).

All protocols and procedures were approved by the authorities of Lower Saxony, Germany, permit AZ 33.9-42502-04-11/0337. Animals were anesthetized with an initial dose of ketamine (10 mg/kg) and xylazine (3 mg/kg) via intramuscular injection. After the initial dose, a tracheotomy was performed followed by intubation, and an exit hole for the air was surgically produced in an abdominal air sac (Schwartzkopff and Brémond, 1963). The animal was then unidirectionally artificially respirated with pure oxygen (∼1 ml/g/min) and 1.5% isoflurane. Depth of anesthesia was constantly monitored via an EKG recording via intramuscular needle electrodes in a wing and in the contralateral leg. Cloacal temperature was monitored and held stable at 41.5°C by a homeothermic blanket system (Harvard Apparatus). The head was firmly held by cementing the skull to a small metal plate connected to a stereotaxic frame (Kopf Instruments, Tujunga, CA, USA). The skull was opened and the cerebellum aspirated to expose the surface of the brainstem for electrode placement, as guided by visual landmarks. We used these landmarks to direct the electrode toward the low-frequency end of the tonotopic axis of NL. Recordings were terminated when sound pressure levels (SPLs) of 70 dB SPL or more were necessary to elicit noticeable responses.

### Electrophysiology and Definition of Recording Types

Recordings were obtained with borosilicate microelectrodes (1.2 mm outer diameter, 0.69 mm inner diameter) filled with either 2 M sodium acetate or artificial cerebrospinal fluid (138 mM NaCl, 2.5 mM KCl, 2.5 mM CaCl_2_, 1 mM MgCl_2_, 10 mM HEPES, 26 mM glucose). Some electrodes were additionally loaded with 5% tracer (10000 MW dextran labeled with Texas Red or biotinylated dextran amine). Typical electrode impedances were between 10 and 20 MOhms. Electrodes were positioned under visual control and then advanced into the brainstem remotely using a piezoelectric motor (Burleigh Inchworm). Recorded potentials were amplified by an Intra 767 electrometer (World Precision Instruments, Sunnyvale, CA, USA). The electrometer was followed by a PC1 spike preconditioner [Tucker Davis Technologies (TDT), Alachua, FL, USA], which amplified and band-pass filtered (300–10000 Hz) the recording and the signal was then passed through a Hum Bug (Quest Scientific Instruments Inc., North Vancouver, BC, Canada) and into a TDT RX6 multifunction processor. Band-pass filtering (50–10000 Hz) and spike detection was carried out after the signal was converted from analog to digital (48 kHz sampling rate, 24-bit resolution) using a custom Matlab (vR2012b, MathWorks, Natick, MA, USA) script.

Single-unit recordings are difficult to obtain in NL and MSO due to the small and variable amplitude of the spikes from the neuronal somata (Scott et al., 2005; Funabiki et al., 2011) and the presence of a strong field potential, the neurophonic (Tsuchitani and Boudreau, 1964; Sullivan and Konishi, 1986). In order to improve unit isolation, we used the loose-patch technique described by Peña et al. (1996). For this, a 5 ml glass syringe was connected to the electrode and a slight positive pressure (corresponding to 1 ml) was maintained while advancing the electrode in order to keep its tip clean. When spikes were detected and the presence of a nearby cell suspected, the positive pressure was released and, if judged necessary, a small negative pressure applied. On many occasions, this technique greatly improved the isolation of spikes. Sub-threshold events were, however, never clearly observed.

The type of recording (single-unit, multi-unit, or neurophonic) was finally defined oﬄine, using the recorded analog data. This also defined the response metric that was analyzed. Traces were classified as spike recordings when they presented consistent action potentials that rose above the background noise and that allowed for triggering using a fixed threshold. Single units were defined as those recordings where an estimated 1% or less of the interspike intervals were smaller than 1 ms (the refractory period). In 11 of 28 single units, the spike sorting script “wave_clus” created by Quiroga et al. (2004) and available from https://www.vis.caltech.edu/rodri/Wave_clus/Wave_clus_home.htm, was used to separate the response of a single unit within a multi-unit spike recording. All responses were tested for a significant neurophonic component using the method described in Köppl and Carr (2008).

### Stimulus Generation and Calibration

All recordings were performed in a double-walled sound-attenuating chamber (Industrial Acoustics Corporation, Winchester, UK). Closed, custom-made sound systems were inserted into both ear canals for controlled stimulation. These systems consisted of small earphones (Yuin PK3 or Sony MDR-E818) and miniature microphones (Knowles TM-3568, EM-3069, or FG-23329), calibrated using a Brüel and Kjaer microphone (B&K 4134, Naerum, Denmark) as the reference. SPLs were then individually calibrated for each ear.

Sound stimuli could be monaural or binaural and were generated separately for both channels by custom-written software and a signal processing device (RX6, TDT). Stimuli were fed from there to the earphones via attenuators (TDT PA5) and headphone buffers (TDT HB7). All stimuli had a total duration of 50 ms, including 5 ms cosine ramps. Recording epochs had a duration of 80 ms with a 120 ms interval between them. The presentation rate was therefore 5 stimuli/s.

### Data Collection Protocols and Analysis

Best frequency (BF), the frequency that evoked the largest response, was determined by presenting a wide range of frequencies at a fixed SPL of 0–20 dB above threshold, as estimated audiovisually. This test was usually run with identical binaural stimulation; in some cases, however, monaural BF curves were run separately. Randomly inserted silent trials were used to determine spontaneous rate.

To obtain an estimate of threshold and response saturation level, monaural rate-level curves were run at a frequency at or close to BF. All ITDs, IPDs, and CDs are shown normalized with negative values always indicating ipsilateral-leading sounds and positive values always indicating contralateral-leading sounds.

### Frequency Threshold Curves (FTCs) and Characteristic Frequency (CF)

Frequency threshold curve (FTC) data were always obtained monaurally. Responses were recorded to a randomly presented matrix of frequencies and SPLs, in steps of typically 100 Hz and 5 dB, and over a range of typically 1 kHz and 50 dB SPL. FTCs were interpolated from this response matrix after smoothing with a locally weighted algorithm (Köppl, 1997). For spike recordings, we adopted a criterion of 20 spikes/s above spontaneous rate as determined from randomly inserted silent trials; this baseline criterion was then adjusted to fit each specific curve. For neurophonic data, the lowest response amplitude that gave a coherent curve was used as a criterion. CF was defined as the frequency at which the criterion response was reached at the lowest SPL, and the corresponding SPL defined the threshold at CF. When possible, we also derived the Q_10__dB_ and Q_40__dB_ (measures of the width of the tuning curve at 10 and 40 dB above threshold), as well as the linear slopes of both FTC flanks between 3 and 23 dB above CF-threshold. CF was our preferred measure of frequency (40 cases, 11 single units). When it was not available, we took BF next (20 cases, 7 single units) and stimulus frequency last (65 cases, 10 single units).

### Best Interaural Time Difference (ITD) and Interaural Phase Difference (IPD)

The range of ITDs tested was ±1 period at or near BF, in steps of 1/10th of a period. The SPL was typically fixed at 0–20 dB above threshold. For spike recordings, the mean rate was derived at each ITD tested; for neurophonic recordings, we determined the average analog amplitude. A criterion that defined significant response modulation with ITD, i.e., the presence of ITD selectivity, was adopted from Köppl and Carr (2008). For spike recordings, the standard deviation and mean spike rate were determined. We then divided the difference between maximum and minimum mean spike rate by the maximal standard deviation observed. Responses were accepted if this value was 1.5 or above. Responses that passed this criterion were fitted with a cosine function at the stimulus frequency to determine best ITD and best IPD. Best ITD was defined as the ITD closest to 0 μs ITD that elicited a maximum response.

For analog recordings, we averaged the analog response waveforms and fitted them with a cosine function at the stimulus frequency. We divided the amplitude of this cosine function by the standard deviation of the averaged waveform multiplied by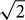. Such an index will have a value between 0 and 1, where 1 indicates a perfect fit and 0 indicates the absence of any component at the stimulus frequency. Recordings were accepted only if this index was larger than 0.7. Best ITD and IPD were determined as above.

### Characteristic Phase (CP) and Characteristic Delay (CD)

Characteristic phase and CD were derived by performing ITD tests at several different frequencies for the same unit or neurophonic site. Three to seven frequencies were used, covering a range of 300–600 Hz around CF. We determined the best IPD for each frequency as described above and entered them into a linear regression of best IPD as a function of frequency (Yin and Kuwada, 1983). The y-intercept of this regression corresponds to the CP, and the slope corresponds to the CD. CP values were collapsed into a single cycle (-0.5 to 0.5). We also adopted a linearity test from Yin and Kuwada (1983).

**FIGURE 1:**
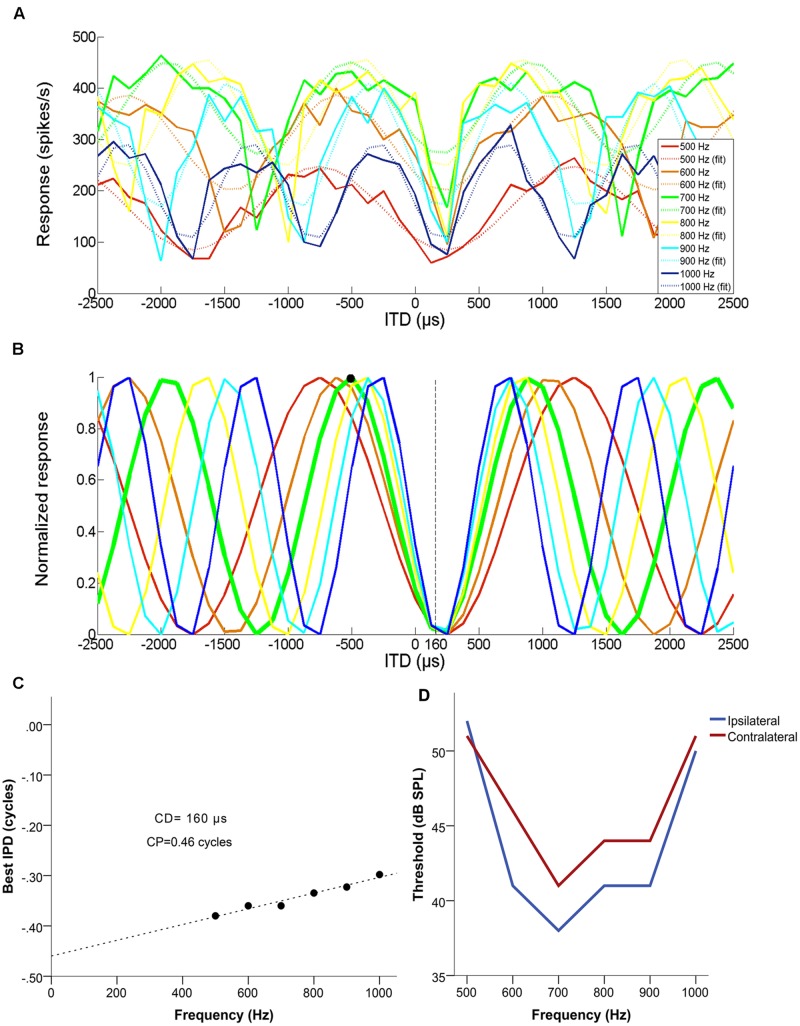
**Example of a set of multiple ITD curves used to determine the characteristic delay (CD) of a single unit with a BF of 700 Hz, a CD of 160 μs contralateral-leading and a CP of 0.46. (A)** Mean discharge rate as a function of ITD, for each of the tested frequencies (500–1000 Hz in 100 Hz steps). Dashed lines represent the cosine fit for each of the curves. **(B)** Normalized cosine fits in relation to the maximum response of each curve for each of the tested frequencies. The response of the neuron at its CF is marked by a thicker green line and the disambiguated best ITD (519 μs ipsilateral-leading) is marked with a black circle. **(C)** The resulting phase-frequency plot, fit with a linear regression. The slope of this fit is the CD and the Y-intercept represents the CP. **(D)** Monaural frequency tuning curves of the example neuron.

**Figure 1** illustrates how CD and CP were determined, for an example single unit with a CF of 700 Hz. **Figure 1A** shows mean discharge rates to five presentations each at different frequencies (500, 600, 700, 800, 900, and 1000 Hz), as a function of normalized ITD (negative = ipsilateral-leading). **Figure 1B** shows the normalized response at each frequency. **Figure 1C** shows best IPD as a function of frequency and the associated linear fit. The CD was used to disambiguate the best ITD of the neuron, by selecting the response maximum closest to the CD. In this example, the neuron had a CP of 0.46 cycles and a CD of 160 μs contralateral-leading. Its CF was 700 Hz (ipsi- and contralateral FTCs shown in **Figure 1D**) and the closest maximum for the corresponding curve fell at 519 μs ipsilateral-leading (679 μs away from the CD) rather than at 910 μs contralateral-leading (750 μs away from the CD). Thus, 519 μs ipsilateral-leading was the unambiguous best ITD (marked in **Figure 1** with a black dot. In this example, it coincided with the peak closest to 0 μs, but this was not always the case. However, all cases where a CD was determined could be disambiguated, i.e., a CP of exactly 0.5 did not occur.

### Labeling and Histology

Labels were placed iontophoretically at selected recording sites by passing a positive DC current through the electrode. Current amplitude and duration varied between 5 and 500 nA, and between 1 and 30 min, respectively. This large variation was due to experimentation to find a set of parameters that resulted in small, specific labels. The set of parameters that yielded the best results was 220 nA for 12 min. At the conclusion of the experiment, the animal was perfused transcardially with 4% paraformaldehyde in phosphate buffered saline (PBS) in order to fix the tissue. The brain was extracted and blocked, and the brain stem was cryoprotected by immersion in 30% sucrose in PBS for 48 h. Sections of 50 μm thickness were cut using a cryostat (Leica CM 1950, Leica Biosystems, Wetzlar, Germany) and mounted in Vectashield. Fluorescent labels were then detected and documented using a Nikon Eclipse 90i epifluorescence microscope with a digital camera attached. Neurobiotin was visualized using standard ABC (Vector Laboratories, Burlingame, CA, USA) and diaminobenzidine protocols on floating sections. After that, sections were mounted and dried on gelatinized slides, counterstained with cresyl violet, dehydrated and permanently coverslipped with DPX.

## Results

**FIGURE 2:**
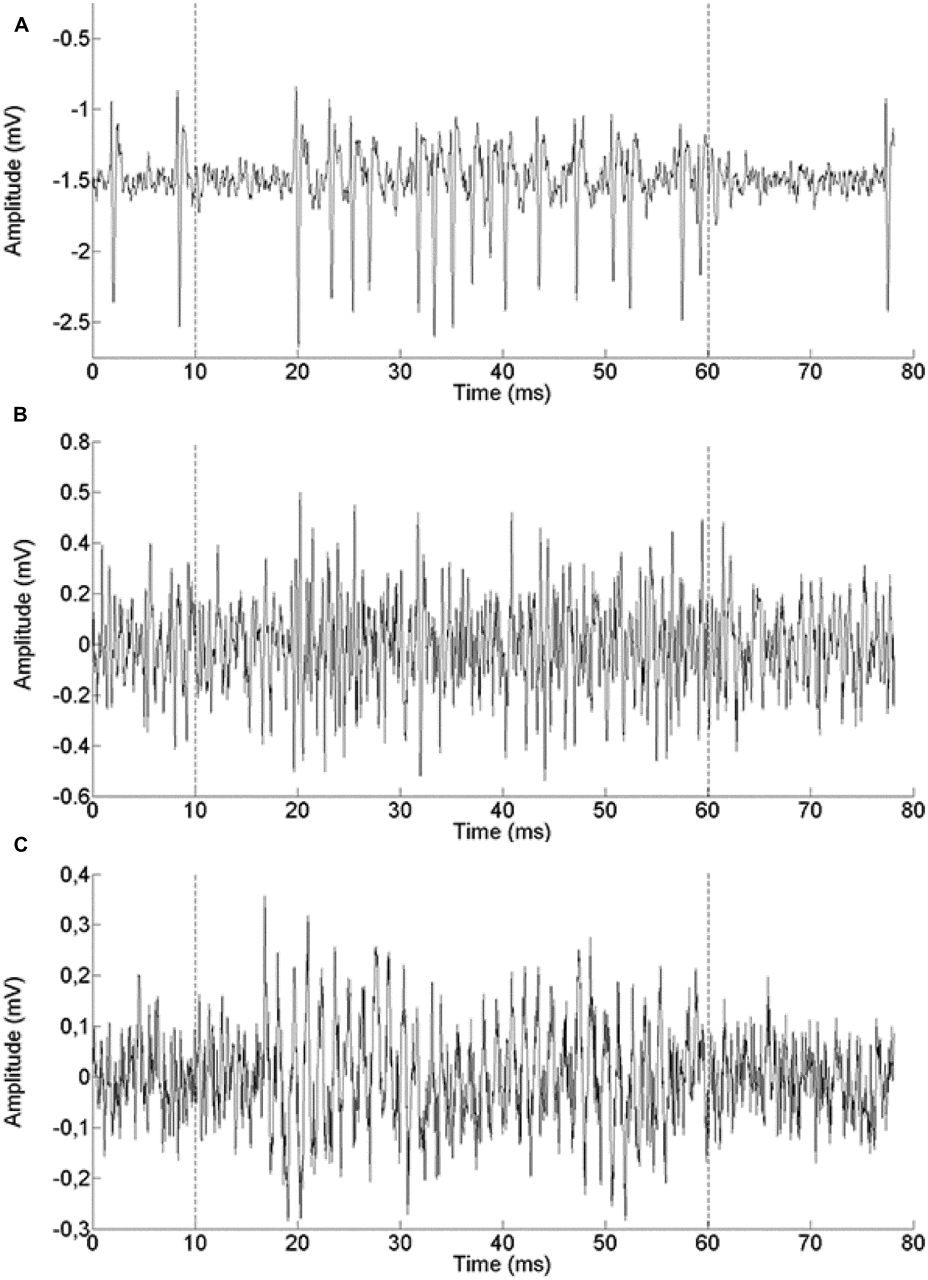
**Example single traces of recordings from a single unit (571 Hz BF, **A**), a multi-unit spike recording (500 Hz BF, **B**) and a neurophonic recording (850 Hz BF, **C**).** The recording epoch lasted 80 ms. The stimulus was presented between 10 and 60 ms (marked by dashed lines).

We report a total of 124 recordings from chicken NL, of which 28 were extracellular single unit recordings (example in **Figure 2A**), 31 were multiunit spike recordings (**Figure 2B**) and 65 were neurophonic recordings (**Figure 2C**). The neurophonic is an extracellular field potential that mimics the acoustic input (Tsuchitani and Boudreau, 1964; Weinberger et al., 1970; Schwarz, 1992). The BFs recorded ranged from 100 to 2800 Hz. A total of 60 out of the 124 recordings (48%) had BFs below 1000 Hz, and the great majority (118 or 95%) fell below 2000 Hz. Chickens have an upper limit of sensitive hearing of 7200 Hz (Hill et al., 2014) and BFs at least as high as 3500 Hz are represented in NL (Köppl and Carr, 2008). The low-frequency bias in our data was intentional and achieved through deliberate targeting. However, no recordings were excluded based on frequency. Six of the recording sites were labeled and confirmed to originate from the cellular layer of NL (data not shown). We aimed to complement the previously published dataset from chicken NL which was high-frequency biased as the low-frequency regions of NL are spatially more compressed and therefore less often hit randomly (Köppl and Carr, 2008). Where appropriate, we will include the previously published data in some of the following figures. The explicit comparison is then made in the last paragraph of the Results.

### Similarity of Neurophonic and Spike Responses

The presence of a neurophonic potential was consistent and easily detected at all tonotopic locations. This neurophonic was well modulated as a function of ITD when the electrode was judged to be inside the cellular region of the nucleus. To test how well neurophonic responses reflected local neural activity, we analyzed 10 cases of paired recordings where spikes and neurophonics were obtained with the same electrode at identical location (**Table 1**). We determined any mismatch between their best IPDs and BFs. Best IPD was chosen (as opposed to best ITD) to account for the difference in stimulus period and thus maximize comparability across sites of very different BF. BFs of the 10 paired recording sites ranged from 400 to 1316 Hz. All pairs had best IPD mismatches of 0.08 cycles or less and best ITD mismatches of maximally 100 μs (**Table 1**). In addition, the best ITD distributions for single units and neurophonics in the entire sample were not significantly different (Kolmogorov–Smirnov test, *p* = 0.573 at frequencies at or below 500 Hz, and *p* = 0.935 at higher frequencies). This suggests that the neurophonic is a good predictor of the response of nearby neurons.

**Table 1:** Comparison of neurophonic and spike recordings (single units shown bold) obtained in close proximity.

	Spike recording		Neurophonic recording				
		
Distance (μm)	Frequency (Hz)	Best ITD (μs)	Frequency (Hz)	Best ITD (μs)	Frequency difference (Hz)	Best ITD difference (μs)	Phase difference (cycles)
**0**	**400**	-**179**	**400**	-**250**	**0**	**71**	**0.028**
0	500	-500	500	-400	0	100	0.050
**0**	**571**	-**612**	**571**	-**700**	**0**	**88**	**0.050**
**0**	**714**	-**472**	**800**	-**375**	**86**	**97**	**0.037**
**0**	**800**	**625**	**800**	**625**	**0**	**0**	**0.000**
**15**	**800**	-**280**	**800**	-**250**	**0**	**30**	**0.024**
0	1000	-480	1000	-400	0	80	0.080
**0**	**1111**	**420**	**1111**	**410**	**0**	**10**	**0.011**
0	1250	-64	1250	0	0	64	0.080
0	1316	-252	1316	-304	0	52	0.068

### Characteristic Frequency (CF), Thresholds and Tuning

**FIGURE 3:**
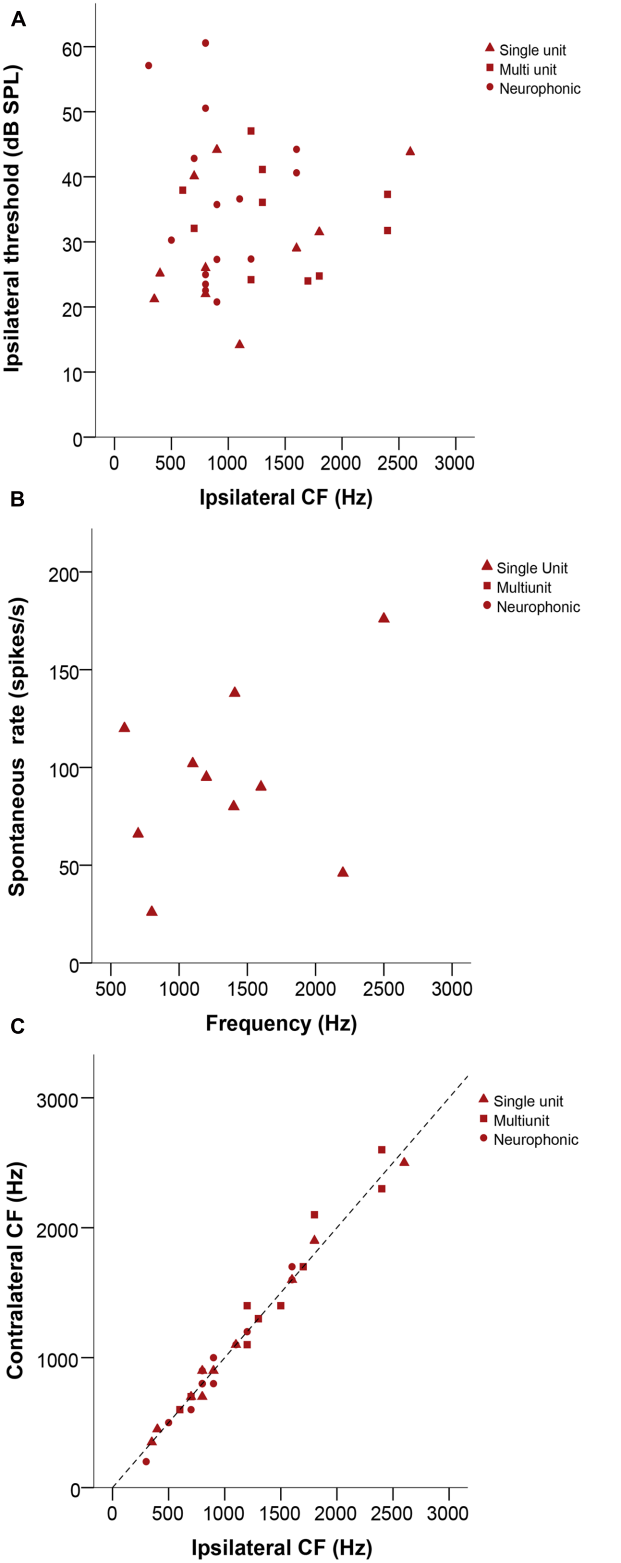
**(A)** Ipsilateral threshold in dB SPL as a function of ipsilateral CF in Hz. **(B)** Plot representing spontaneous rate in spikes/s as a function of frequency (Hz). **(C)** Ipsilateral versus contralateral characteristic frequencies (CFs) in Hz. The dashed line represents X = Y. In all panels, triangles represent single-unit recordings, squares represent multi-unit recordings and circles represent neurophonic recordings.

Characteristic frequency (CF) values ranged from 200 to 2600 Hz. Thresholds were variable, ranging from 13 to 61 dB SPL (**Figure 3A**). Sharpness of tuning, as measured by Q_10__dB_, ranged from 1.2 to 13, with slightly lower values at low frequencies. The spontaneous rate of single units ranged from 26 to 138 spikes/s (**Figure 3B**). Ipsi – and contralateral thresholds and Q_10__dB_ values were not significantly different (Wilcoxon signed-rank test, *p* = 0.182 and 0.627, respectively, *n* = 37 for threshold, *n* = 23 for Q_10__dB_, all recording types). There were also no significant mismatches between the CFs obtained with ipsi- and contralateral stimulation (Wilcoxon signed-rank test, *p* = 0.532, *n* = 35, **Figure 3C**).

### Best ITD and IPD Distribution

Due to the nature of ITD sensitivity in narrowly frequency-tuned neurons such as those in NL, the response modulation is cyclical, which means that, within our usual ITD testing range of ±1 period of the stimulus frequency, we will find two peaks of maximal response, one of which will lie in the ipsilaterally leading range of ITDs and the other in the contralaterally leading range. This causes the best ITD and IPD values to be ambiguous, since it cannot be resolved which of the two peaks corresponds to the time difference between the neuron’s binaural inputs. For our analysis, we defined the peak closest to zero ITD as the best ITD. However, only additional measurements, such as taking responses at several different frequencies and determining the CD and CP (see Materials and Methods) can truly resolve this ambiguity. Among our 124 recording sites, 23 were tested for ITD selectivity at several stimulus frequencies, and had their CD and CP determined (example shown in **Figure 1**). The relationship between best IPD and frequency can usually be expressed using a linear regression (example shown in **Figure 1C**). The slope of this equation is the CD and the y-intercept is the CP. All but one of the 23 cases were linear according to the criteria developed by Yin and Kuwada (1983), at a significance level of 0.05 or smaller; 18 cases were linear at a significance level of 0.005 or smaller.

**FIGURE 4:**
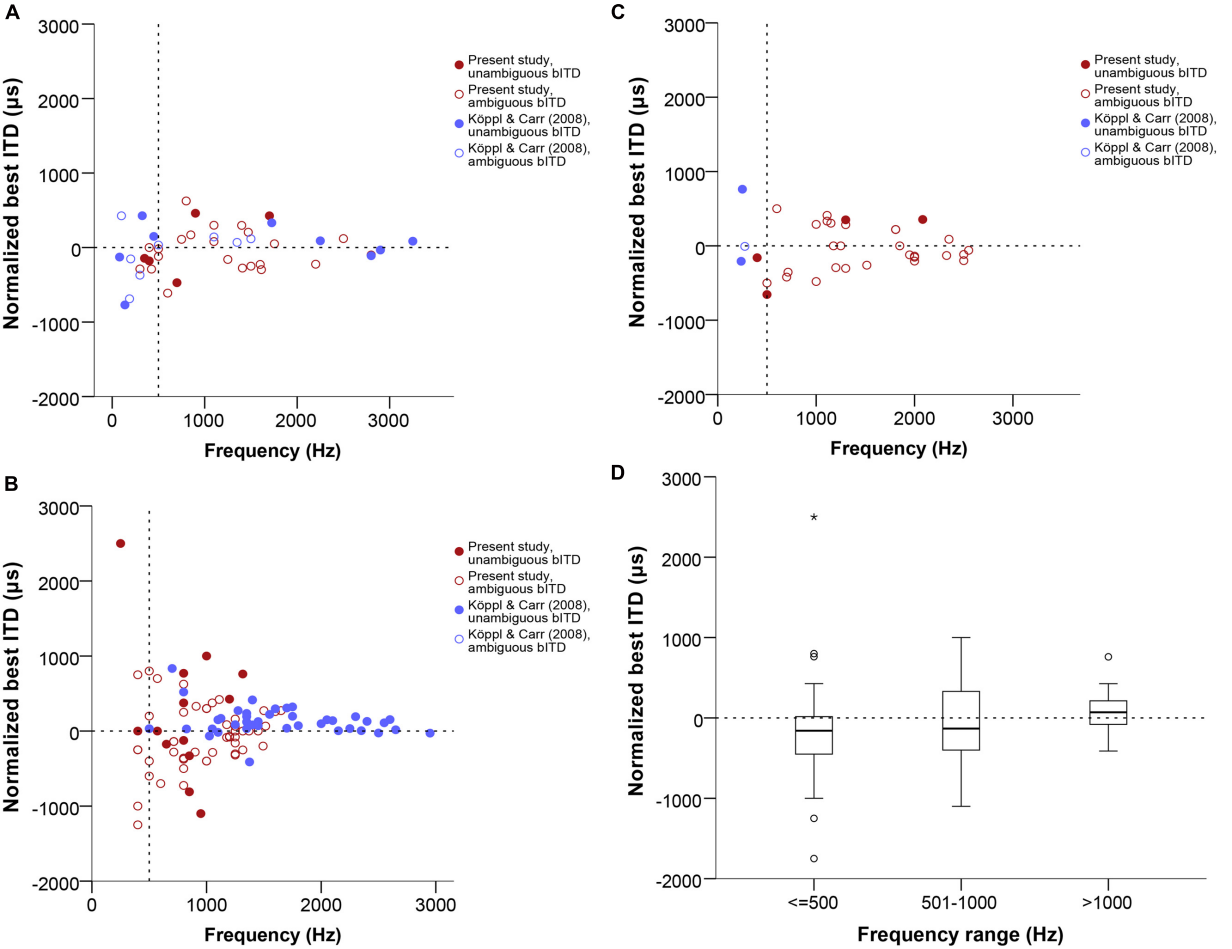
**Normalized best ITD in μs as a function of stimulus frequency (Hz) near or at CF, separate for single units **(A)**, multi units **(B)**, and neurophonics **(C)**.** In order to easily visualize and compare data across all three panels the extreme outlier at -4312 μs is not shown. Red data points are data from this study, blue data points are data from Köppl and Carr (2008). Filled and open symbols represent ambiguous and unambiguous bITDs respectively. **(D)** Box plot of the best ITD distribution of all datasets and recording types in μs at different frequency ranges, expressed in Hz. Solid bars represent the median, boxes represent the interquartile range. Whiskers represent the maximum and minimum of the distribution, circles and stars mark outliers. In all panels negative values represent ipsilateral-leading sounds and positive values represent contralateral-leading sounds. The horizontal dashed line marks the acoustic midline at 0 μs ITD and the vertical dashed line marks the 500 Hz position.

In **Figure 4**, data labeled as ambiguous represent cases where ITD-sensitivity was tested at just one frequency and the best ITD was defined as the response peak closest to zero. Data labeled as unambiguous represent best ITD-values that were re-defined, if needed, as the response peak closest to the CD.

Best ITD values ranged from 0 to 2500 μs contralaterally leading and to -4312 μs ipsilateral-leading. Data were fairly symmetrically distributed around zero ITD, i.e., the number of neurons recorded with contralateral and ipsilateral best ITDs was similar (67 ipsilateral versus 46 contralateral, 11 recordings with a best ITD of exactly 0 μs). The ITD ranges covered in ipsilateral and contralateral space were also similar and increased with decreasing frequency (**Figures 4A–C**). There were no differences between the different types of recordings shown in separate panels of **Figures 4A–C** (Kruskal–Wallis test, *p* = 0.909). **Figure 4D** summarizes the medians and interquartile ranges of best ITD values across all recording sites, but separated according to three different tonotopic regions. Medians always fell very close to zero ITD, reflecting the already mentioned symmetrical distribution around zero ITD.

**FIGURE 5:**
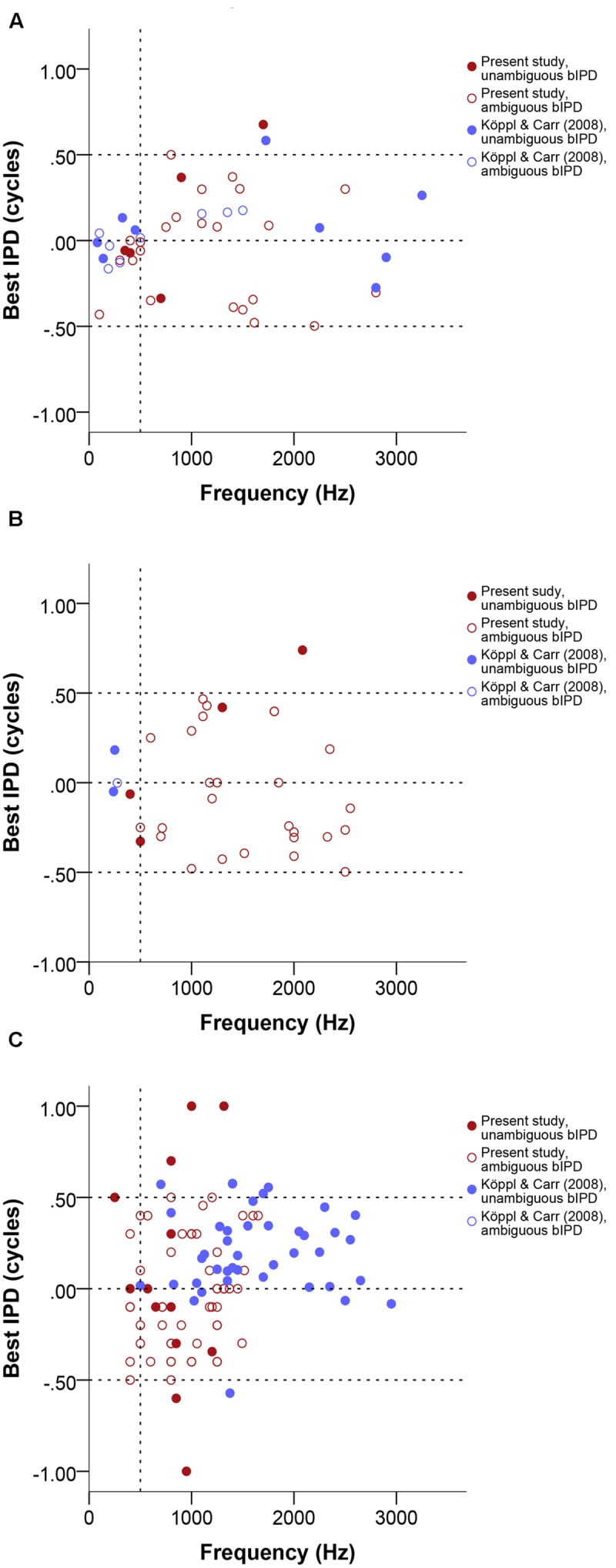
**Normalized best IPD in cycles as a function of stimulus frequency (Hz) near or at CF, separate for single units **(A)**, multi units **(B)**, and neurophonics **(C)**.** Red data points are data from this study, blue data points are data from Köppl and Carr (2008). Filled and open symbols represent ambiguous and unambiguous bIPDs respectively. Negative values represent ipsilateral-leading sounds and positive values represent contralateral-leading sounds. Dashed horizontal lines mark the acoustic midline at 0 μs ITD and the pi limit at ±0.5 cycles. A vertical dashed line marks the 500 Hz position.

Best IPD values generally ranged from -0.5 to 0.5. Single- and multi-unit data clustered around zero at frequencies below 500 Hz. Neurophonics and data above 500 Hz did not cluster around specific values at different frequencies and their distribution was frequency-independent (**Figure 5**). We found only 7 of 124 best IPD values from the present study (red circles in **Figure 5**) outside the pi limit, a range corresponding to half the period of the stimulus frequency and equivalent to the maximum best ITDs that can be generated using phase delays (Vonderschen and Wagner, 2014).

For 7 of 22 cases where CD and CP were also determined (32%), the disambiguated response maximum, i.e., best ITD, was not the one closest to zero; these appear as unambiguous data points outside the pi-limit in **Figures 5A–C**. In the majority of these (five of seven), the peak closest to zero was ipsilateral-leading while the peak closest to the CD value was contralateral-leading; in the other two cases the reverse was true. Even among those cases where the peak closest to zero was the closest to the CD, there were four cases in which the laterality of best ITD and CD differed (best ITD ipsilateral, CD contralateral). This could occur because the CD often did not coincide with a response peak (see next section). Ambiguous and unambiguous ITDs were significantly different (Kolmogorov–Smirnov test, *p* = 0.01).

### Characteristic Phase and Characteristic Delay

**FIGURE 6:**
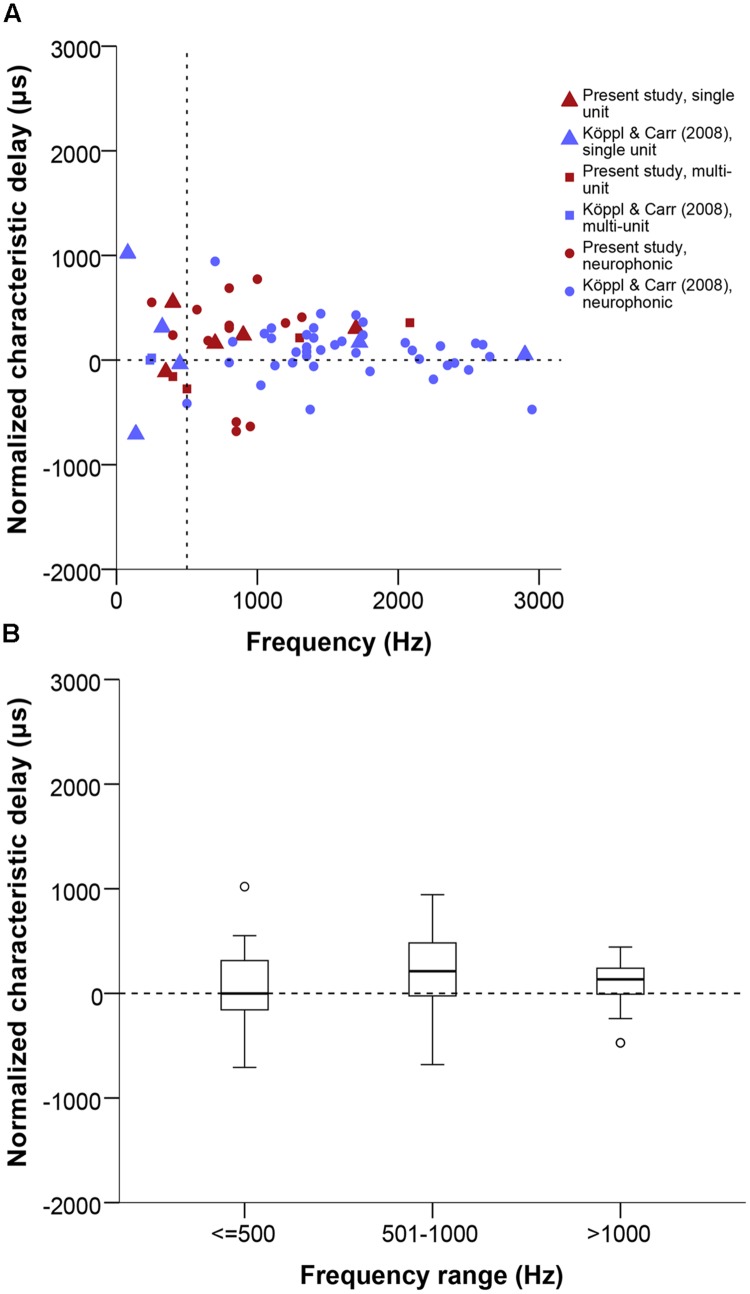
**(A)** Normalized CD in μs as a function of stimulus frequency (Hz). Triangles represent single unit recordings, squares represent multiunit recordings and circles represent neurophonics. The acoustic midline at 0 μs ITD is marked by a horizontal dashed line and a vertical dashed line marks the 500 Hz position. Red data points are data from this study, blue data points are data from Köppl and Carr (2008). **(B)** Box plot of the CD distribution of all datasets and recording types in μs at different frequency ranges, expressed in Hz. Solid bars represent the median, boxes represent the interquartile range. Whiskers represent the maximum and minimum of the distribution, circles and stars mark outliers. Negative values represent ipsilateral-leading sounds and positive values represent contralateral-leading sounds.

We analyzed the range and distribution of CD and CP values of the 22 tested cases that showed a linear phase-frequency relation (see previous section). CD values ranged from 773 to -681 μs (**Figure 6**). The general distribution appeared shifted toward contralateral-leading values when compared to best ITD (**Figures 4D and 6B**). However, this was not supported when testing for a difference between best ITD and CD for the restricted sample where both measures were taken (Wilcoxon test, *p* = 0.492).

**FIGURE 7:**
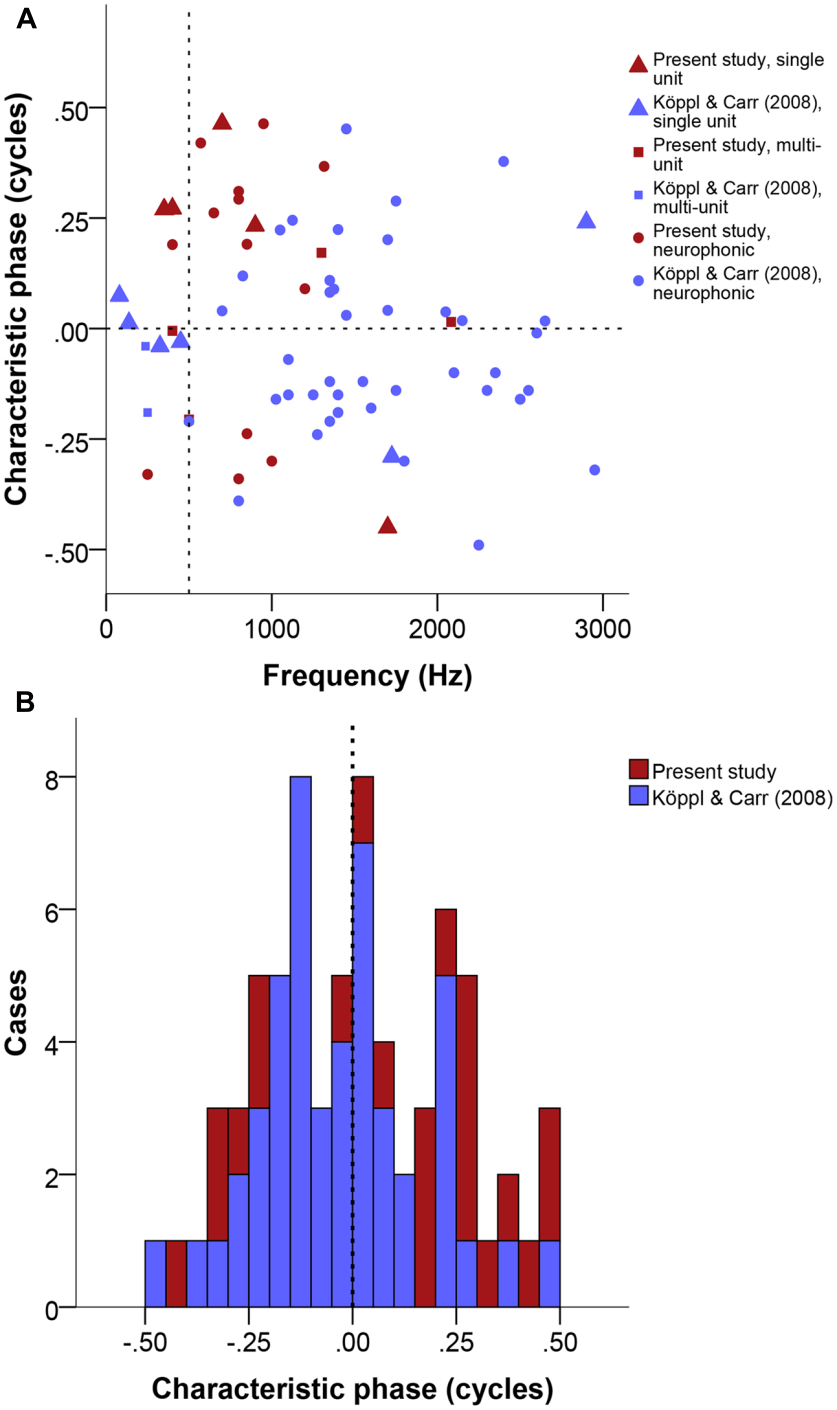
**Characteristic phase (CP) normalized to a single cycle (±0.5). (A)** CP in cycles as a function of stimulus frequency (Hz). Triangles represent single unit recordings, squares represent multiunit recordings and circles represent neurophonics. The horizontal dashed line marks the acoustic midline at 0 μs ITD and the vertical dashed line marks the 500 Hz position. **(B)** Stacked histogram of CP normalized to one cycle. Bins are 0.05 cycles wide. The dashed line marks the zero position. Data shown in red are from the present study, data shown in blue from Köppl and Carr (2008).

Phase-frequency relationships were quite diverse (**Figure 7A**). Pure time-delay systems like the Jeffress model are expected to show close to 0 or to 1. Other values indicate that there is some phase-delay contribution, i.e., delays that vary with frequency (Vonderschen and Wagner, 2014). Three of our 22 cases showed a CP close to 0 or 1 (within ± 0.15), thus indicating a CD close to a peak in the ITD curves. Eleven cases showed intermediate CPs (in the range of | 0.15–0.35|), indicating that the CD occurred at some point along the slopes of the ITD curves. Lastly, 8 of 23 had CP values close to 0.5 (within ± 0.15), which indicates that the CD occurred near a trough in the ITD curves (example in **Figure 1**). Overall, the CP distribution was near normal (**Figure 7B**), with a median at 0.036 CP did not depend on frequency (**Figure 7A**).

### Slope Midpoints

To address the question whether the peaks or slopes of the ITD-response curves better cover the naturally heard ITD range, we also determined slope midpoints as an indicator of the point of maximal sensitivity to ITD. For each recording site, we determined the midpoint of the slope of the ITD sensitivity curve that fell closest to the acoustic midline. This was calculated as ¼ of the stimulus period toward zero ITD from the best ITD. For example, for a best ITD of +1000 μs at 1000 Hz, the slope midpoint would correspond to +750 μs, while for a best ITD of -1000 μs the slope midpoint would correspond to -750 μs. In this kind of analysis, the direction of the slope becomes significant and thus unambiguous determination of best ITD. Therefore, we only included disambiguated data here.

**FIGURE 8:**
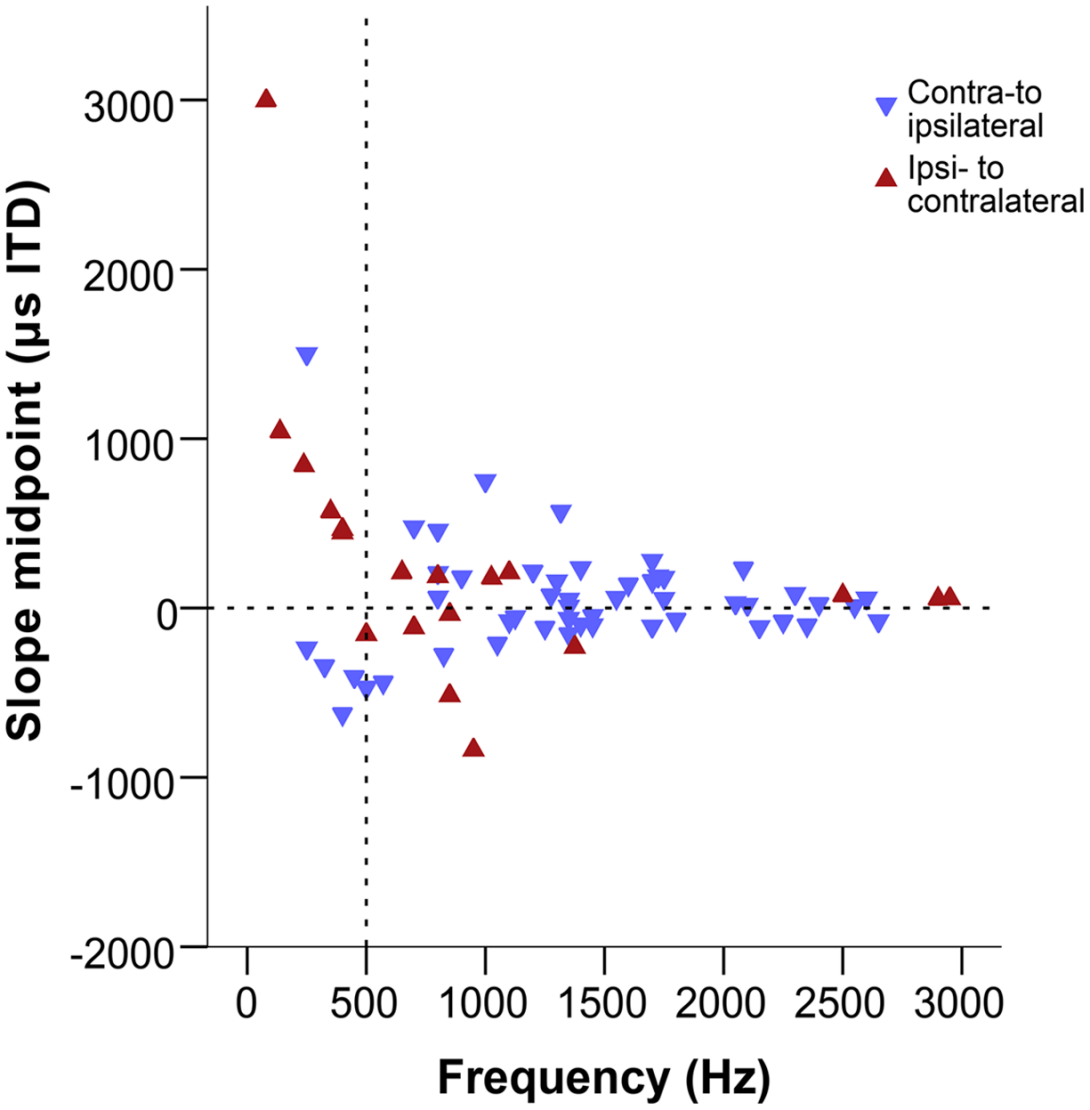
**Slope midpoints of the ITD-response functions from the combined data of this study and Köppl and Carr (2008).** Negative values represent ipsilateral-leading sounds and positive values represent contralateral-leading sounds. Red triangles represent ipsi- to contralateral downward slopes, blue triangles represent contra-to ipsilateral downward slopes, i.e., both types of triangles point in the downward direction of the slope. The horizontal dashed line marks the acoustic midline at 0 μs ITD and the vertical dashed line marks the 500 Hz position.

**Figure 8** shows the slope midpoints, coded according to the direction of the slope relative to the peak. If slopes were centered on the acoustic midline, data points will cluster near the zero line on the graph. This is consistent with the distribution at frequencies above ∼500 Hz. There, slope midpoints were homogeneously distributed across a range of values that decreased with increasing frequency (**Figure 8**). Furthermore, most slopes showed a consistent direction, from a peak in contralateral space toward ipsilateral (indicated by the orientation of triangles). However, the distribution of slope midpoint values for sites with a BF below ∼500 Hz showed a change: most of the slope midpoints now corresponded to large ITD values and a nearly equal number of slopes traversed the acoustic midline in both directions, from a peak in ipsilateral space to a trough in contralateral space and vice versa.

### Comparison with Previously Published Data of Chicken NL

As explained above, the present recordings were deliberately biased toward lower BFs and intended to complement the previously published data of Köppl and Carr (2008). **Figures 4–7** display both datasets together. Other than frequency, we did not detect any systematic biases that would preclude pooling those data. Indeed, the main difference is a methodological one, in that the present dataset is larger but many recording sites were not as completely characterized, i.e., there was a higher proportion of ambiguous best ITDs. Ambiguous and unambiguous ITDs showed significantly different distributions in both the new dataset alone and both datasets combined (Kolmogorov–Smirnov test, *p* = 0.01 for the present dataset, *p* < 0.001 for both datasets combined) while unambiguous data were similar between both datasets (Kolmogorov–Smirnov test, *p* = 0.024). This highlights the value of determining best ITD unambiguously to avoid sampling biases.

## Discussion

We present a comprehensive dataset of electrophysiological recordings of the responses to ITDs of neurons from the low frequency regions of chicken NL. These data allow for a more direct comparison between chicken and mammalian recordings of ITD-sensitive neurons, which thus far covered different frequency ranges. We will focus on features of our data that are novel and suggest that the classic Jeffress-type place code of ITD holds in modified form at higher frequencies, while at frequencies below several 100 Hz the data are not consistent with any current model of ITD coding.

### Neurophonic as a Proxy Measurement for Single Unit Responses

Both the NL of archosaurs and the MSO of mammals present neurophonic responses with clear ITD sensitivity (Wernick and Starr, 1968; Sullivan and Konishi, 1986; Schwarz, 1992; Carr et al., 2009). ITD sensitivity is also a defining characteristic of neurons in those nuclei. However, the precise source of the neurophonic response is not yet clear and seems to vary depending on the specific anatomical organization (Kuokkanen et al., 2010; McLaughlin et al., 2010; Goldwyn et al., 2014). We compared neurophonic and spike responses taken in the same location or in very close proximity, and none of the pairs showed a best phase difference larger than 0.08 or a best ITD difference larger than 100 μs between spike and neurophonic recordings (for details see **Table 1**). In addition, the general distribution of best ITDs showed no significant difference between single units and neurophonics. A similarly good correspondence between neurophonic and single unit responses has repeatedly been shown in owl, chicken and alligator (Köppl and Carr, 2008; Carr et al., 2009; Funabiki et al., 2011; Palanca-Castan and Köppl, 2015). We thus consider the neurophonic response as a reasonable proxy for single unit responses.

### Evidence for Different Types of Input Delays

We observed an abundance of responses with non-zero CP, which means that the CD occurred at some point on the slope of the response functions, rather than at a peak. Such behavior is consistent with phase delays contributing to the binaural inputs. Phase delays may be generated by a variety of physiological mechanisms such as timed inhibition (Brand et al., 2002), cochlear delays through mismatched inputs from the two ears (Yin and Kuwada, 1983; Shamma et al., 1989; Day and Semple, 2011) or synaptic properties (Jercog et al., 2010; Franken et al., 2015). Most recently, it has been suggested that even the acoustic inputs themselves could underlie non-zero CPs (Benichoux et al., 2015).

The distribution of best ITDs appeared homogeneous at any given frequency, which is consistent with a time-delayed, Jeffress-like system. In contrast, a two-channel model, based entirely on phase delays, predicts some degree of clustering around a single specific ITD value that should decrease with increasing frequency (McAlpine et al., 2001; Brand et al., 2002; Vonderschen and Wagner, 2014). Physiological data from birds and crocodilians as well as from mammals often show an intermediate behavior, where the total range of best ITDs decreases with increasing frequency, albeit without any clustering to specific ITD values (review in Joris and Yin, 2007; Pecka et al., 2008; Carr et al., 2009; Bremen and Joris, 2013). This was also true for the chicken NL.

We also analyzed our data regarding the pi-limit, a theoretical limit for the best ITD distribution of a phase-delay system (Vonderschen and Wagner, 2014) and equal to half the period of the stimulus frequency. This limit exists because the cyclical nature of phase differences means that, e.g., a contralateral-leading phase delay of 0.6 cycles cannot be differentiated from an ipsilateral-leading phase delay of 0.4 cycles. Pure time-delay systems like the Jeffress model do not have this theoretical limitation. The maximum ITD that a time delay is able to compensate depends on the specific limitations of the delay mechanism, e.g., the maximum physical length difference between axons arriving from the ipsilateral and contralateral ears. Therefore, best ITDs should be distributed across the naturally occurring range, irrespective of the frequency band. In practice, selecting the best ITD closest to zero from responses to a single test frequency means that measurements will always fall within the pi limit if they are not disambiguated by additional tests. In addition, a recent modeling study suggests that a similar frequency dependence of best ITD range may result for systems based on time delays and phase delays alike (Fontaine and Brette, 2011). Therefore, a larger dispersion of best ITDs at lower frequencies is not a strong argument against a delay-line system à la Jeffress. Our dataset contained several points outside the pi-limit, both in the best ITD (after disambiguation) and CD distributions, which suggests the presence of a time-delay mechanism. The period of the frequencies we probed is long enough so that points outside of the pi-limit were also outside the natural ITD-range for the chicken. Finally, our data provided no support for a stereausis model. This model (Schroeder, 1977; Shamma et al., 1989) is based on cochlear delays, which are caused by monaural inputs arriving to the coincidence detectors with a slight CF mismatch. There were no significant or systematic CF mismatches, in either the present or the previously published data (Köppl and Carr, 2008).

In summary, the chicken NL shows evidence for a mixed contribution of potentially several sources of input delays. Strong and long-standing evidence for frequency-invariant time delays via axonal delay lines comes from both anatomy and physiology (Overholt et al., 1992; Köppl and Carr, 2008). Our data suggest that additional, frequency-dependent phase delays play a significant role. The strongest evidence is the broad range of CP values, which is difficult to reconcile with a pure time-delay system. It is important to emphasize that several sources of delays need not be incompatible or in conflict. Indeed, a similar combination of frequency-invariant time delay and frequency-dependent phase delays was found to best explain the responses of neurons in the gerbil MSO (Day and Semple, 2011). This does not mean that the sources of such delays are necessarily similar in gerbil and chicken.

### Relationship of ITD Representation in NL to the Chicken’s Natural Range

In a two-channel model of ITD coding, many best ITD values are predicted to fall outside the naturally heard range of the animal (Harper and McAlpine, 2004), while the Jeffress model predicts all values to fall within that range. In the chicken, the physical separation of the ears yields a prediction of ±75 μs for its natural range of perceived ITDs (Hyson et al., 1994). However, in practice, the presence of internal coupling between the middle ears via the interaural canal makes this range larger, with a more pronounced effect at lower frequencies (Calford and Piddington, 1988; Larsen et al., 2006). The internally coupled middle ears extend the effective physiological range of the chicken close to 200 μs at 800 Hz, and possibly even larger at lower frequencies (Hyson et al., 1994; Fischer and Seidl, 2014) but due to a lack of measurements, the exact values for very low frequencies remain unknown.

Both best ITD and CD values were distributed over a range that extended beyond ±200 μs, and thus is probably broader than the natural range of the chicken. The homogeneity speaks against a two-channel model of ITD coding, while the broad distribution range is inconsistent with a classical Jeffress-type model. In contrast, the slope midpoints were much more restricted in distribution at frequencies above ∼500 Hz. At lower frequencies, the distribution was not homogeneous. Instead, all values fell outside the likely natural range and no values occurred at ITDs close to the acoustic midline.

### ITD Coding Changes Across the Tonotopic Range in the Chicken

Two features in our data are not consistent with a classical Jeffress model. Firstly, a broad range of best ITDs and CDs that extended beyond the likely natural range of the chicken. Secondly a large number of non-zero CPs that point to the additional contribution of a phase delay system. The position of slope midpoints at higher frequencies was, however, more restricted to the likely natural range of the chicken and homogeneously distributed within it. This suggests that, in accordance with previous predictions (Harper and McAlpine, 2004; Hyson, 2005; Fischer and Seidl, 2014) and reports (Harper et al., 2014) the chicken may use a slope code representation as opposed to a peak code. Importantly, this is not in conflict with a topographical representation that, indeed, was already shown for the higher-frequency regions in the chicken NL (Köppl and Carr, 2008). This points to a modified Jeffress-type place code as proposed by Hyson (2005), where each ITD is represented by the border between clusters of maximally firing units and minimally firing units across an array of NL neurons. It is important to note that peak and slope coding are not mutually exclusive, and both systems could be used to extract information about different features.

However, our data suggests that this system does not extend to low frequencies of several 100 Hz. In this range, slope midpoints fell far from the acoustic midline, with (a) no homogeneous distribution, (b) no values close to 0 μs ITD, and (c) no consistent slope direction across the acoustic midline. All of these contradict the concept of a systematic array of slopes covering the natural range. It remains unknown whether there is a topographic organization of ITDs at these low frequencies. Our data for low frequencies also did not support an ITD coding strategy according to the two-channel model. In particular, the presence of response slopes crossing the acoustic midline in both directions in each NL would destroy any correlation of the relative hemispheric activities with ITD (Grothe et al., 2010). Furthermore, the slope midpoints, i.e., the most sensitive regions of the responses, curiously spared the representation of frontal space. Thus far, our data for the very low frequencies of chicken NL do not support any current model of ITD coding.

This is different to the barn owl, where we recently showed that the properties of NL neurons remained consistent with a Jeffress model down to frequencies of several 100 Hz (Palanca-Castan and Köppl, 2015). Different representations of azimuthal space in chicken and barn owl could be a reflection of the necessities of their respective ecological niches similarly to the space covered by visual fields (Martin, 2007). Indeed, Heffner and Heffner (1992) and Heffner (1997) found a consistent relationship between the field of best vision and sound localization acuity in which animals with smaller fields of best vision (usually associated with predators) had higher acuity. While the extraordinary sound localization capabilities of the barn owl are well-documented (Knudsen et al., 1979), it remains unknown how well chickens can localize sounds. Given that the chicken may not need the localization precision that the owl does, the constraints on the neural circuits need not be as narrow.

## Conflict of Interest Statement

The Editor Catherine E. Carr and the Reviewer Jose Luis Peña declares that, despite collaborating on an article with Christine Köppl in 2014, the review was conducted objectively. The Reviewer Paula Tuulia Kuokkanen declares that, despite collaborating on an article with Christine Köppl in 2013, the review was conducted objectively. The authors declare that the research was conducted in the absence of any commercial or financial relationships that could be construed as a potential conflict of interest.
